# Role of internal tide mixing in keeping the deep Andaman Sea warmer than the Bay of Bengal

**DOI:** 10.1038/s41598-020-68708-6

**Published:** 2020-07-20

**Authors:** A. K. Jithin, P. A. Francis

**Affiliations:** 1 Indian National Centre for Ocean Information Services (INCOIS), Ministry of Earth Sciences, Government of India, Hyderabad, India; 2 Department of Meteorology and Oceanography, Andhra University, Visakhapatnam, Andhra Pradesh India

**Keywords:** Physical oceanography, Climate sciences, Hydrology

## Abstract

Vertical profiles of temperature obtained from various hydrographic datasets show that deep waters (below 1,200 m) in the Andaman Sea are warmer (about 2 °C) than that of the Bay of Bengal. As a result, the biochemical properties in the deep waters also exhibit significant differences between these two basins. Higher temperature in the deep waters of Andaman Sea compared to the BoB had been widely attributed to the enclosed nature of the Andaman Sea. In this study, we show that strong tidal energy dissipation in the Andaman Sea also plays an important role in maintaining the higher temperatures in the deep waters. Dissipation rates inferred from the hydrographic data and internal tide energy budget suggests that the rate of vertical mixing in the Andaman Sea is about two-orders of magnitude larger than that in the Bay of Bengal. This elevated internal tide induced vertical mixing results in the efficient transfer of heat into the deeper layers, which keeps the deep Andaman Sea warm. Numerical experiments conducted using a high-resolution setup of Regional Ocean Modelling System (ROMS) further confirm the effect of tidal mixing in the Andaman Sea.

## Introduction

Temperature distribution in the deep ocean plays an important role in regulating the deep ocean circulation, water mass formation, distribution of chemical properties as well as the distribution of marine organisms including benthic life forms^1–4^. In addition, a good understanding on the distribution of temperature, both near the ocean surface and interior ocean is essential to decipher the response of the ocean to climate change^5^. Some recent studies have shown that effects of climate change are more pronounced in the deep ocean and in the marginal Seas^6^.

**Figure 1:**
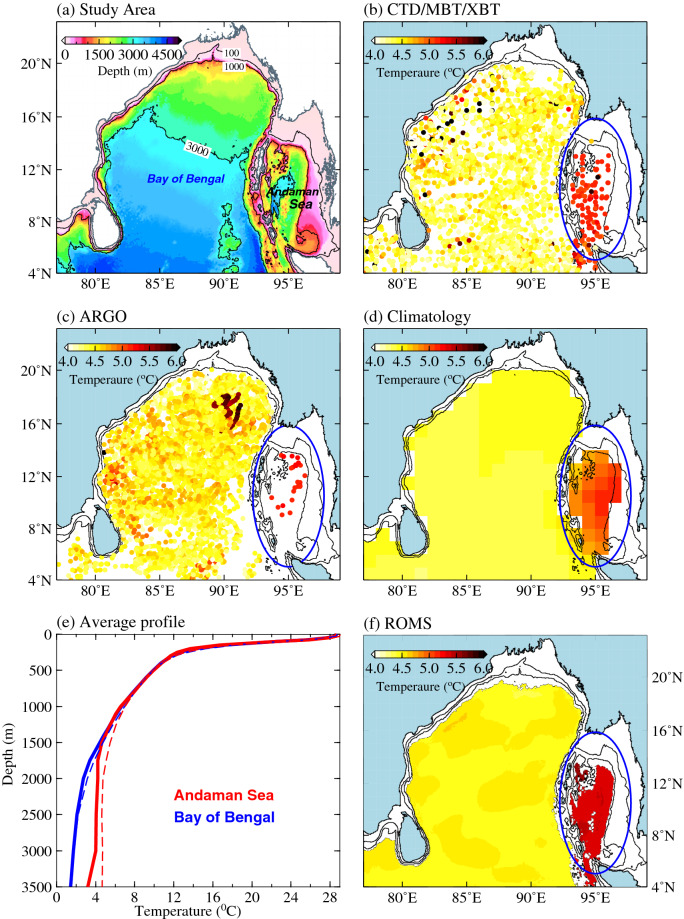
(**a**) Study area with bathymetry. (**b**) Temperature at 1,500 m in the Bay of Bengal and Andaman Sea from (**a**) all historical hydrographic observations, (**b**) Argo profiles and (**c**) revised WOA2009 climatology for the Indian Ocean^27^. Blue ellipse represents the Andaman Sea, where the temperature is higher than the Bay of Bengal. (**e**) Average vertical profile of temperature in the Bay of Bengal (blue) and Andaman Sea (red) from CTD/XBT/MBT observation (think lines) and from the model (dashed lines). (**f**) Spatial distribution of temperature at 1500 m in the model (ROMS). Note that a positive bias of 0.25 °C has been removed from the model simulations at 1,500 m.

Andaman Sea (AS) is a semi-closed marginal sea located in the north-eastern part of the tropical Indian Ocean which is partly-isolated from Bay of Bengal (BoB) by Andaman-Nicobar (AN) Ridge (Fig. 1a). About 85% of the AS is shallower than 2,500 m and maximum depth is about 4,200 m. AS is connected to BoB through the shallow passages such as Preparis Channel in the north (250 m deep), and Ten Degree Channel (800 m deep) and Great Channel (1,800 m deep) in the south. Low sea surface salinity in the AS and BoB due to the large freshwater influx from the rivers makes this region unique from other oceanic basins^7^. Surface layers in the BoB and AS are characterized by uniform temperature along the same latitudinal belt, which gradually decreases from equator to poleward. However, there exists a striking difference between the temperature distribution in the deep levels of BoB and AS, that the AS is warmer (about 1–2 $^{\circ }\hbox {C}$) than that of the BoB below about 1,000 m (Fig. 1a). This temperature difference exists in all the seasons as reported in many previous studies^8–10^. This temperature difference between deep AS and BoB is anticipated due to the enclosed nature of AS, which restricts the water exchange with BoB and AS. However, the role of other oceanic processes in keeping the deep AS warm are not yet studied well.

In general, the deep ocean is not significantly affected by surface processes in the short timescales to a large extent and it is ventilated mainly by the deep ocean circulation and diapycnal mixing^11^. Since the AS is partially isolated from BoB, it is possible that the warm deep water remains trapped for a longer period due to the small lateral inter-basin water exchange in the deep layers. In such conditions, the renewal time of deep waters can be more than hundreds of years like those in the South China Sea^12^ and Japanese Sea^13^. However, the estimation of $^{230}$Th activity and measurements of radiocarbon ($^{14}$C) in the deep waters of the AS suggested that the bottom waters get replaced very rapidly with a renewal time less than 6 years^14,15^. In addition, the lateral exchange of water between AS and BoB is about 2 Sv with large mass transport across the Great Channel, which is as deep as 1,800 m^14^. Therefore, deep waters in the AS are not stagnant and bottom waters are not completely trapped. Hence, factors other than trapping warm water due to the partially enclosed nature of the AS also can contribute to the observed temperature difference. For example, it is possible that since the Andaman Sea basin is an active geothermal region with a younger oceanic crust, the high heat flow from the bottom in this region may contribute to the warming of bottom layers^16–18^. However, none of the observed hydrographic data in this region show evidence for such heat flow and associated warming in the bottom levels^15,19^

Another possibility is the transfer of heat from the upper layers to bottom due to strong interior mixing^11^. Major source of mechanical energy required for the vertical mixing in the interior ocean is the breaking of internal gravity waves^20^. Most of the internal wave activity in the ocean primarily occur at near-inertial frequencies and tidal frequencies, where the former is generated by the wind action on the ocean surface and later is by tidal flow over the topographic features^21,22^. AS is well known for the presence of large internal waves generated by tidal forcing, known as internal tides^23^. The AN Ridge is the main source of internal tides in this region^24,25^. Internal tides generated along the AN Ridge radiates into both BoB and AS. In an earlier study, Wang et al.^26^ showed that internal tide induced mixing plays an important role in the vertical and horizontal distribution of water mass properties such as temperature and salinity and their transformation in the South China Sea. Since AS is characterised by strong internal tide activity, it is possible that internal tides play a significant role in regulating the temperature distribution in the AS also. Hence, we examine the role of internal tide energy dissipation and associated vertical mixing in maintaining the observed temperature difference between the deep regions of AS and BoB in this paper.

The rest of the paper is organized as follows. "Data and methods" section describes the data and numerical experiments. Results are discussed in third section. Summary and Discussion are given in fourth section.

## Data and methods

### Observations

In this study, we use temperature profiles from all available historical hydrographic surveys in the BoB and AS to examine the temperature distribution in this region. These data sets include measurements using Conductivity-Temperature-Depth (CTD), Expendable Bathythermograph (XBT), Mechanical Bathythermograph (MBT), Drifting Buoys (DRB), and Undulating Oceanographic Recorders (UOR). The main source of these hydrographic data is National Oceanographic Data Center (NODC) and detailed description as well as the quality assessment of these historical data sets are discussed in Chatterjee et al.^27^. We analyze 48,386 such temperature profiles which have vertical coverage deeper than 1,500 m. We also analyze temperature profiles from Argo floats for the period 2002–2016. There are about 7250 CTD profiles from Argo floats, which have data coverage deeper than 1,500 m (https://www.nodc.noaa.gov/argo/). The temperature profiles from the revised climatology data sets described by Chatterjee et al.^27^ are also used in this study (https://did.nio.org/climatology/temperature_and_salinity_climatology).

### Model simulations

Simulations from a high-resolution model based on Regional Ocean Modelling System (ROMS) configured for the BoB and AS are used to study the tidal energetics and their role in the observed temperature difference^24,28,29^. ROMS is a three-dimensional, non-linear, finite difference, free surface hydrostatic model developed by Rutgers University^30^. Primitive equations in ROMS are formulated in terrain-following vertical coordinate system and finite curvilinear orthogonal Arakawa-C grid in horizontal. The domain of the present model configuration extends from 77$^{\circ }$ to 99$^{\circ }$E and 4$^{\circ }$ to 23$^{\circ }$N, which covers the entire BoB and AS (Fig. 1a). Horizontal resolution of the model is about 2.3 km (1/48$^{\circ }$) and 40 sigma level in vertical. The initial and boundary conditions for this model are obtained from a basin scale model for the Indian Ocean with a resolution of 9 km (1/12$^{\circ }$) described in Effy et al^31^. Tidal forcing in this model is implemented using the amplitude and phase values of 10 tidal constituents (M$_2$, S$_2$, N$_2$, K$_2$, K$_1$, O$_1$, P$_1$, Q$_1$, Mf and Mm) derived from a global barotropic tidal solutions, TPXO 7.0^32^. Model is also forced with 6-hourly atmospheric fields (analysis) obtained from the Global Forecast System (GFS) configured by the National Centre for Medium Range Weather Forecast^33^. Horizontal mixing along the geopotential surfaces is represented by harmonic mixing scheme and bulk parameterization scheme is used for the computation of air-sea fluxes^34,35^. The sub-grid scale vertical mixing in the model is represented by a nonlocal K-Profile Parameterization (KPP) scheme^36^.

The integration of this high-resolution model was initialized on 01 June 2010 using the initial and boundary conditions extracted from a Indian Ocean Model with a horizontal resolution of 1/12$^{\circ }$ and simulation extends upto March 2016^37^. Model has taken about two and half years to become stable after the initialization and hence the simulation from 1 January 2013 has been used for this study. Hourly model simulations for a 1-year period (January–December 2013) are used to estimate the internal tide generation, propagation and dissipation in this region. TUGOm Tidal ToolBox^38^ is used to extract tidal signals from model output. Validation of the internal tides simulated by this model against observations from several Acoustic Doppler Current Profilers (ADCPs) deployed along the east coast of India^24^, and the data from the RAMA (Research Moored Array for African–Asian–Australian Monsoon Analysis and Prediction) moorings in the BoB and the deep ocean moorings from AS as well as the internal tide signals obtained from satellite altimetry data^39^ have shown that the model simulates the observed features of internal tides in this region very well. Further details of the model configuration and validation of the subtidal circulation simulated by this model can be seen in Jithin et al.^28^ and Francis et al.^29^. In this study, specific sensitivity experiments using this model with and without tidal forcing are also carried out to examine the effect of tidal forcing on the temperature distribution in this region. Details of these numerical experiments and their results are given in "Impact of tidal forcing on the observed temperature distribution" section.

### Estimation of internal tide energetics

Assuming that the tendency and advection of internal tide energy are small (*E*) over a tidal period, dissipation (*D*) of internal tides is estimated as

1 $$\begin{aligned} \nabla \cdot { \mathbf {F}} + D \ = \ C \end{aligned}$$

here *C* is the barotropic to baroclinic energy conversion and *F* is the depth-integrated baroclinic energy flux^40,41^. Here *C* is estimated as

2 $$\begin{aligned} C =\ < \nabla H \cdot {\mathbf {U}_{bt} \ p'_{b}}> \end{aligned}$$

where *dh*/*dx* and *dh*/*dy* are the slopes of bathymetry in the east-west and north-south directions, U$_{bt}$ is the barotropic tidal current and $p_{b}'$ is the baroclinic pressure perturbation at the bottom at tidal period^42,43^. Depth-integrated baroclinic energy flux (*F*) in the model is estimated using the expression,

3 $$\begin{aligned} \mathbf {F}=\int \limits _{-H}^0 < \mathbf {u'}(z) \ p'(z)> dz \end{aligned}$$

where $u'$ and $p'$ are the baroclinic velocity fluctuation and baroclinic pressure anomaly respectively. Angle brackets ($<>$) indicate average over a tidal period^44^. Detailed discussion on the internal tide energy budget and its estimation can be found in Jithin et al.^24,39^.

## Results

### Differences in the subsurface temperature between the BoB and AS: observations and simulations

In order to obtain a more comprehensive picture of the subsurface temperature difference between the BoB and the AS, we examined the bottom temperature distribution in the two basins by combining all the available data from historical hydrographic surveys, Argo profilers and climatological data sets (Fig. 1b–d). Results show that there are no noticeable differences between AS and BoB in the temperature distribution in the upper 1,200 m water column. However, deep waters (below 1,200 m) in the entire AS is about 1–2$^{\circ }\hbox {C}$ warmer than the rest of the basin. In addition, based on the World Ocean Atlas climatology (WOA, https://www.nodc.noaa.gov/OC5/WOA09/pr_woa09.html) the observed temperature in deep AS is warmer (for example, about 1$^{\circ }$C at 1,300 m) than the global average temperature of the deep tropical ocean. The comparison of average temperature profiles between the observation and model simulation suggests that the model simulates the observed temperature difference reasonably well (Fig. 1e). However, simulated temperature in the AS (average over the domain) is warmer compared to observation at some depths, which results in a larger temperature difference between the basins in the model. For instance, the temperature difference is about 1$^{\circ }$C at 2,000 m depth in the climatological data, but the difference in the model is about 2$^{\circ }$C at 2,000 m). In an earlier study, Sarma et al.^8^ analyzed the hydrographic data collected along the eastern and western parts of the AN Ridge and showed that there exists a difference of about 2$^{\circ }$C in the temperature between the BoB and AS at 2,000 m. Similarly, Sewell^45^ reported that the temperature difference is about 1.7$^{\circ }$C at 1,829 m. This suggests that the temperature difference between the deep BoB and AS in the model simulations is not unrealistic (see Supplementary 1) and this difference probably due to the averaging of relatively small numbers of observed profiles in the AS. Spatial distribution of 1-year average temperature from ROMS simulations at 1,500 m presented in Fig. 1f also shows that the model simulates this observed temperature difference realistically, though the model has a warm bias of about 0.25$^{\circ }$C throughout the basin. As discussed in the introduction, internal tide induced mixing could play an important role in the observed temperature distribution. Hence, the internal tide energetics and their role in the observed temperature distribution in the deep AS and BoB are examined in the subsequent sections.

### Internal tide energy dissipation in the BoB and AS

**Figure 2:**
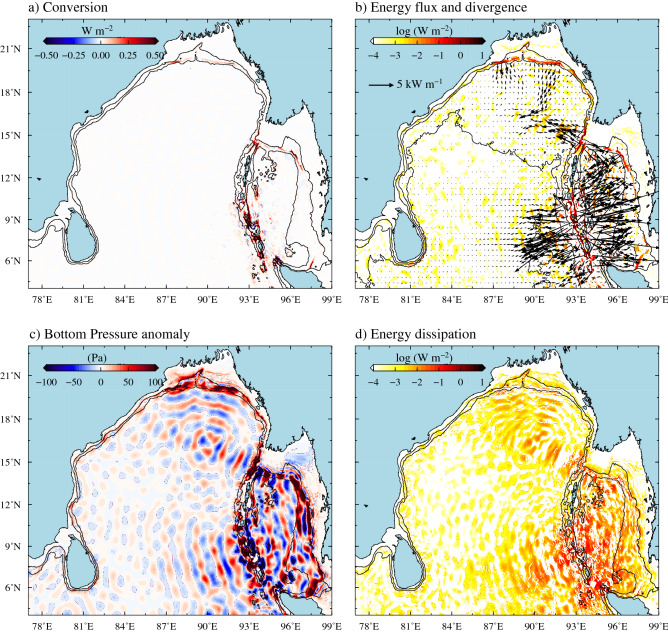
(**a**) Barotropic to baroclinic M$_2$ energy conversion rate. (**b**) Depth-integrated baroclinic M$_2$ energy flux (vectors) in the BoB and flux divergence (filled colour). (**c**) Bottom pressure anomaly of M$_2$ internal tides. (**d**) Depth-integrated energy dissipation rate of M$_2$ internal tides.

Tides in the BoB and AS are semidiurnal in nature and the largest tidal constituent is M$_2$ (12.42 h), known as the principal lunar semidiurnal constituent^46^. Semidiurnal S$_2$ is the second largest tidal constituent and its spatial variability is similar to that of M$_2$. The ratio of maximum tidal elevation of other major tidal constituents such as S$_2$, N$_2$, K$_1$, and O$_1$ with respect to M$_2$ in this region is 44%, 16%, 11% and 6% respectively^46^. Therefore, here we mainly discuss the energetics of M$_2$ internal tides since they explain a large fraction of internal tide generation in this region. The barotropic to baroclinic energy conversion rate and the energy flux for the M$_2$ tides in the BoB and AS derived from the numerical model are shown in Fig. 2. The potential generation sites of internal tides in this region are the continental slopes in the head of the bay, AN Ridge, and continental slopes in the northeastern and southeastern parts of the AS. In addition, rough topographic features and seamounts within the deep AS also favours the internal tide generation (Fig. 2a).

The tidal energy budget estimates using the model simulations show that the barotropic M_2_ tides loses about 65.5 GW of tidal energy in the model domain, in which 71.5% ($\sim$ 46.5 GW) is lost by dissipation due to bottom friction and rest of the energy is converted as baroclinic energy (19.1 GW), which gives rise to generation of internal tides. About 79.5% (14.7 GW) of this energy conversion occurs along the AN Ridge and the internal tides generated over the ridge radiate into both BoB and AS (Fig. 2b). Bottom pressure anomaly of M$_2$ internal tides clearly shows the radiation of internal tides into the BoB and AS from AN Ridge (Fig. 2c). Recently, Jithin et al.^24,39^ have reported that the internal tides, which radiate westward from the AN Ridge, propagate over large distances (1,000–1,400 km) across the BoB. They further showed that a part of these internal tides that shoal onto the continental margins of the east coast of India get further transmitted on to the inner shelf and eventually dissipate there. The internal tides radiate eastward from the AN Ridge dissipate inside the Andaman Sea. Our estimates show that a significant fraction (57% $\sim$ 10.4 GW) of the total internal tide energy dissipation occurs in the AS, which constitute only about 25% of the total area of BoB and AS (2,139,717 km$^2$). As a result, the depth-integrated energy dissipation rate (W m$^{-2}$) is about two orders of magnitude higher in the AS compared to that in the BoB (Fig. 2d). This large internal tide energy dissipation over a small region can lead to larger diapycnal mixing in the AS compared to BoB.

The most appropriate way to verify the differential rate of mixing is to compare the observed vertical diffusivity rates from microstructure profilers in both the regions. However, in situ observations of full-depth turbulent mixing rates are not available in the BoB or in the AS to examine the rates of vertical mixing in these regions. Hence, we examine the spatial variability of internal tide induced mixing in this region using the rate of vertical diffusivity ($K_{v}$) inferred from hydrographic profiles as well as estimated from modelled internal tide dissipation using parameterization scheme. In addition, the effect of tidal forcing is further examined by a set of specific sensitivity experiments using the ROMS configuration.

### Differential rate of diapycnal mixing in the BoB and AS

**Figure 3:**
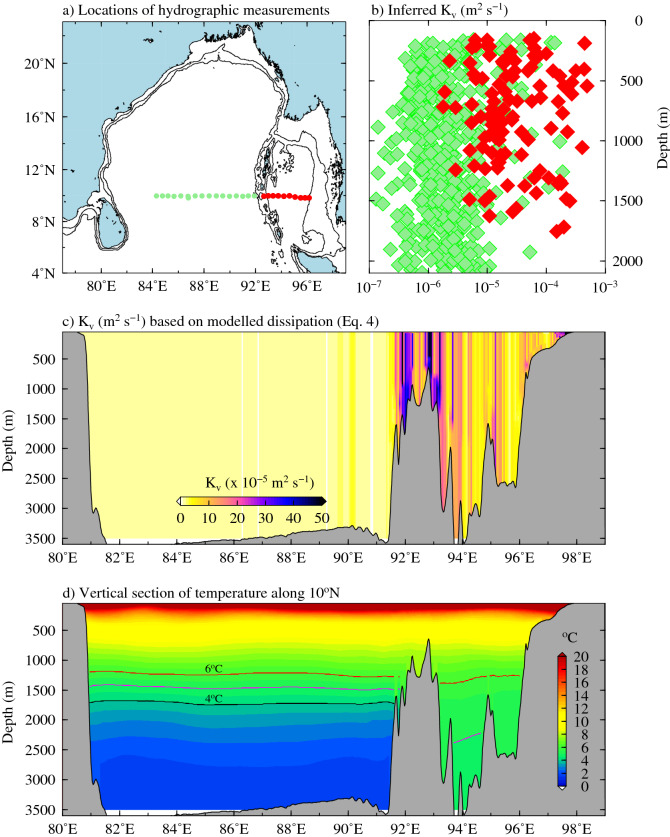
(**a**) Locations of Hydrographic casts in the BoB (green) and AS (red) used to calculate the vertical diffusivity ($K_v$) by Kunze^47^. Black contours represent 100 and 1,000 m bathymetric contours. (**b**) Vertical profiles of eddy diffusivity along 10$^{\circ }$N estimated from these hydrographic casts from Kunze^47^. (**c**) Vertical diffusivity estimated based on LS02 internal tide parameterization scheme (Eq. 4) and (**d**) modelled vertical distribution of temperature along the 10$^{\circ }$N.

The vertical profiles $K_{v}$ estimated by Kunze^47^ from hydrographic casts based on vertical strain (finescale parameterization based on vertical strain) along 10$^{\circ }$N in the BoB and AS are shown in Fig. 3a, b. Earlier studies noted that the spatial variability of the $K_{v}$ values inferred using this method are consistent with those obtained from the microstructure observations^20,47^. It may be clearly seen from Fig. 3b that the $K_{v}$ values are about two-orders of magnitude larger in the AS ($10^{-5}$–$10 ^{-3} \,\hbox {m}^2 \,\hbox {s}^{-1}$) compared to that in the BoB ($10^{-7}$–$10^{-5}\, \hbox {m}^2 \,\hbox {s}^{-1}$). This confirms the hypothesis that the rates of vertical mixing in the AS is higher compared to that in the BoB and the spatial variability is consistent with internal tide energy dissipation shown in Fig. 2d.

To get a more comprehensive picture, we also estimated the values of $K_{v}$ using an internal tide parameterization scheme proposed by St. Laurent et al.^48^[hereafter LSJ02]. In this study we adopted a modified version of LSJ02, which was used to parameterize the internal tidal mixing in the Indonesian Seas^49^ and the Yellow Sea^50^, given as

4 $$\begin{aligned} {\begin{matrix} K_v &{} =\Gamma \frac{qE(x,y)}{\rho \int N^{2} dz } + K_0, \ when \ dN/dz <0 \\ &{} = \Gamma \frac{qE(x,y)}{\rho N \int N^{2} dz } + K_0, \ when \ dN/dz >0 \end{matrix}} \end{aligned}$$

where $\Gamma$ is the mixing efficiency, (considered here as 0.2), *q* is the local dissipation efficiency (0.3) and *E*(*x*, *y*) is the barotropic to baroclinic energy conversion. Here, *N* is the buoyancy frequency, $\rho$ is the density, *x* and *y* is the longitude and latitude coordinates. Background diffusivity ($K_0$ $=$ 10$^{-5}$ m$^{2}$ s$^{-1}$) is assumed to be a constant. In LSJ02 scheme, *qE*(*x*, *y*) represents a fraction (about 30%) of energy (which is converted from barotropic to baroclinic) dissipated in the generation sites. The rest of the energy, which radiates away eventually dissipates in remote regions, are not considered in this parameterization scheme. In this study, we replaced the *qE*(*x*, *y*) in Eq. (4) with the dissipation rate (*D*) obtained from energy budget calculation (see "Data and methods") as suggested by Liu et al.^50^. It may be noted that the observed spatial variability of the interior mixing has a strong dependency with the distribution of internal tide dissipation in the ocean and this relationship has been confirmed with the observational data in previous studies^20,51^. Hence, even though there may be biases in these estimates compared to actual values of $K_{v}$, the spatial variability of internal tide induced mixing will be mostly consistent.

Longitude-depth section of $K_{v}$ estimated based on Eq. (4) is shown in Fig. 3c. It may be noted that higher values of $K_v$ are seen over the AN Ridge. Several previous studies using observations and model simulations have reported increased vertical mixing over the submarine ridges in many other parts of the world ocean^52^. It can be seen from Fig. 3c that the $K_{v}$ values in the AS are about one-two orders of magnitude ($1 \times {10}^{1} - 1 \times 10^{2}$) higher than that in the BoB. This spatial difference is also consistent with the inferred $K_{v}$ values obtained from Kunze^47^. Hence the higher rates of vertical mixing in the AS can lead to efficient vertical distribution of heat over the water column compared to BoB. Thus, bottom layers in the AS are in warmer equilibrium state than BoB. The vertical section of temperature along the 10$^{\circ }$N shows that the regions of warm deep layers (especially below 1,200 m) in the AS matches well with high values of $K_{v}$ (Fig. 3d).

### Impact of tidal forcing on the observed temperature distribution

**Figure 4:**
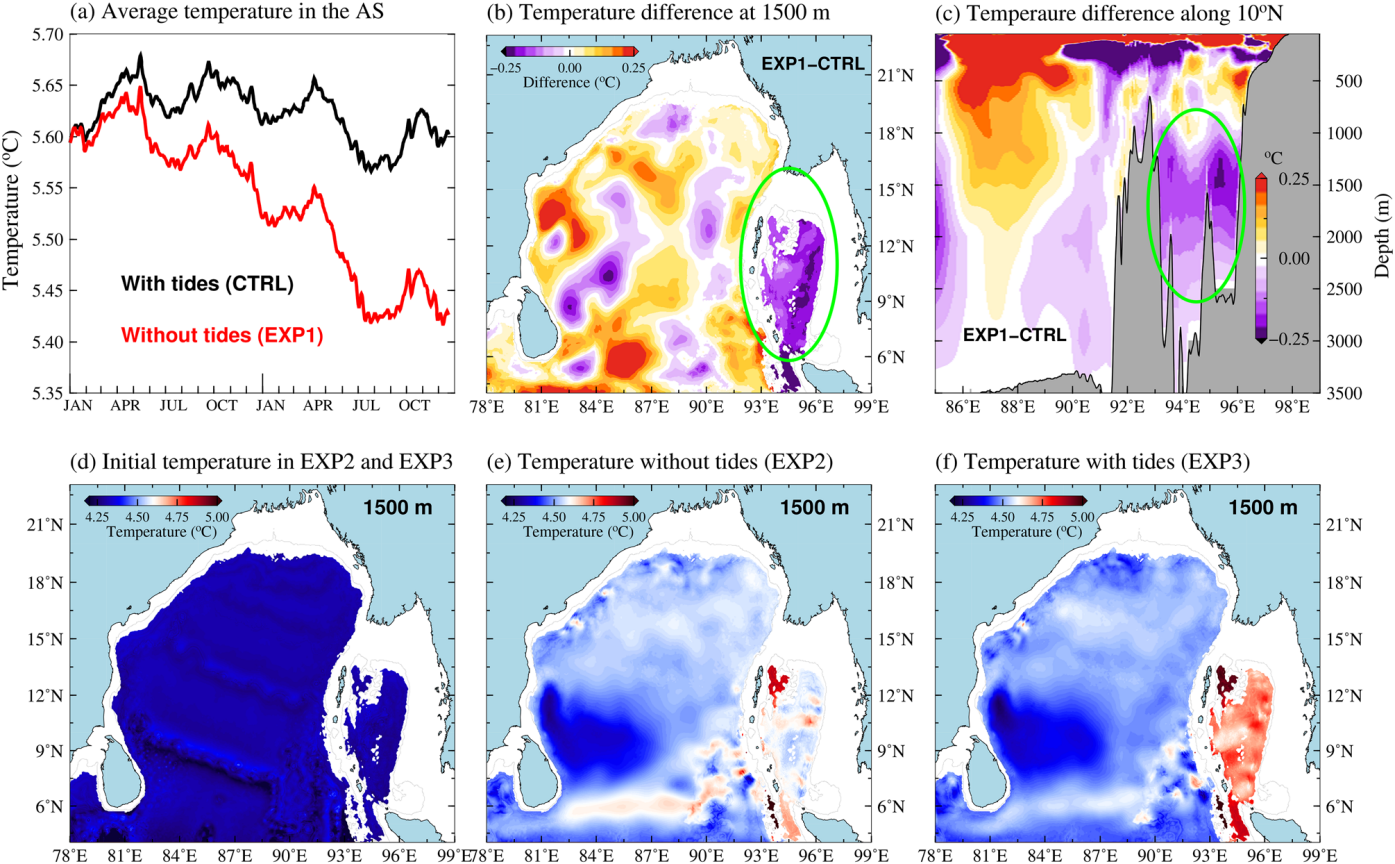
(**a**) Evolution of average temperature at 1,500 m in the AS with (CTRL) and without tidal forcing (EXP1) for a 2-year period (2013–2014). (**b**) Temperature difference between the model simulations without and with tidal forcing at 1,500 m (EXP1-CTRL) after 2 year (December 2014). (**c**) Temperature difference between the model simulations without and with tidal forcing along 10$^{\circ }$N (EXP1-CTRL) after 2 years. Green circle represents AS, where the temperature cooling occurs when the tidal forcing is stopped. (**d**) Temperature distribution at 1,500 m in the initial conditions (01 January 2013) of EXP2 and EXP3. (**e**) Temperature at 1,500 m after 2 years of model simulation with tidal forcing (EXP2). (**f**) Temperature at 1,500 m after 2 years of model simulation without tidal forcing (EXP3).

The effect of tidal mixing on the subsurface temperature distribution in the deep BoB and AS is further examined by conducting a series of sensitivity experiments using the same ROMS configuration described in "Model simulations" section. In the first experiment, the model simulations are carried without applying the tidal forcing for the period of 1 January 2013 to 31 December 2014 (EXP-1). The initial conditions for this experiment are taken from the high-resolution ROMS model simulations with tides started from 1 June 2010 (CTRL run). Therefore, the deep AS is already warmer compared to deep BoB in the initial state of EXP1. In addition, realistic atmospheric and boundary conditions are applied in both the model simulations. Though the internal tide mixing is not explicitly parameterized in ROMS, a fraction of the vertical diffusion of heat due to internal tide dissipation will be accounted for by the vertical shear term in the KPP parameterization scheme^36^. The temperature evolutions in the AS at 1,500 m depth in the model simulations with and without tidal forcing are shown in Fig. 4a. It may be seen that the mean temperature in the deep AS is maintained nearly uniform throughout the period of simulations, except their seasonal modulation when forced with tide (CTRL). Note that deep waters (1,500 m) in the AS becomes slightly warmer during the inter monsoon period (April−May and October−November period). This seasonal variation could be due to the advection of warm water into the Andaman Sea as reported by Chatterjee et al.^53^ as a response to the eastward flowing Wyrtki Jet^54^ in the equatorial Indian Ocean.

It is interesting to note from Fig. 4a that the deep AS started cooling gradually when the tidal forcing is stopped (EXP1). After 2 years of model simulations without tidal forcing (December 2014), the average temperature in the deep AS (at 1,500 m) cooled by about 0.25$^{\circ }$C. This suggests that the heat transfer from the upper layers to bottom layers by vertical mixing has reduced significantly in the absence of the tidal forcing. In contrast to the BoB region, the spatial distribution of temperature difference between the simulations of EXP1 and CTRL shows a cooling trend everywhere in the deep AS (Fig. 4b). However, deep layers in the BoB do not show any spatially consistent trend of cooling or warming when the tidal forcing is removed. We also carried out model simulations for a period of 10 years (2005–2015) with and without tidal forcing to examine the consistency of these results (Supplementary 2). Results from these longer model simulations also suggest that the deep waters in the AS becomes cooler in the absence of tidal forcing (by about 0.25$^{\circ }$C after 10 years). It may be noted that the surface layers, especially in the AS and near the AN Ridge, become warmer while it becomes cooler near thermocline in these regions in the absence of tidal forcing (Fig. 4c). This suggests a reduction in the heat transfer across the thermocline region in the absence of internal tide mixing. Similar response of tidal mixing in the upper layers in the AS was also noted by Jenson et al.^55^.

It is possible that the warming or cooling in the deep layers of AS in the earlier experiment (EXP2) may have a dependency to pre-existing spatial distribution of temperature in the initial condition. In order to eliminate this preconditioning of the ocean, the initial temperature and salinity profiles at every model grid are replaced with the profiles of domain-averaged values (Fig. 4d) in the second (EXP2) and third experiments (EXP3). The difference between EXP2 and EXP3 is that the tidal forcing is absent in the former but present in the latter. The evolution of subsurface temperature values from these experiments are shown in Fig. 4e, f. It may be seen that, without the tidal forcing, the temperature at 1,500 m does not change significantly in the AS and there is no coherent trend in temperature change in the BoB as well as AS. Interestingly, the deep AS becomes warmer (about 0.2$^{\circ }$C at a depth of about 1,500 m) after 2 years of model simulation with tidal forcing (EXP3). In addition, the increasing trend in temperature in the AS is coherent in the entire AS. This further illustrates the importance of tidal energy dissipation and associated mixing in keeping the deep AS warm.

## Summary and discussion

Observations presented in this study as well as previous studies show that the deep AS is about 1–2$^{\circ }\hbox {C}$ warmer than that of the BoB below 1,200 m^8–10^. The cause of this temperature difference was mainly attributed to the enclosed nature of AS in the earlier studies. Our investigation shows that elevated internal tide induced vertical mixing also plays an important role in the formation of warm deep waters in this region. The AN Ridge, which separates these semi-enclosed tropical ocean regions as BoB and AS, is the major source of internal tides in this region. Estimates show that a significant fraction (57% of the domain integrated conversion) of the internal tide energy dissipation occurs in the AS, which constitute only about 25% of the total area of BoB and AS. Therefore, a large fraction of internal tidal energy dissipates over a smaller region. Our analysis shows that dissipation rates of internal tides in the AS are about two-orders of magnitude larger than BoB. Hence the higher vertical mixing rates induced by internal tides cause a higher rate of heat transfer from upper layers to the deep waters of AS. Numerical experiments carried out in this study further confirm the role of internal tide mixing in maintaining the warmer temperature in deep AS. In situ observations of vertical mixing using instruments like microstructure profilers can give a better picture of this differential rate of mixing in this region.

Temperature in the deep ocean plays a significant role in determining the distribution of biogeochemical properties of the ocean. In an earlier study, Sarma et al.^8^ reported that oxygen concentration in the deep AS (below 1,200 m) is about 70–120 $\upmu$mol kg$^{-1}$ lower than the BoB. They also noted that, in addition to small exchange of deep waters, higher temperature, which decreases the solubility of gases also has contributed to the observed difference in the biogeochemical properties between AS and BoB. Sharma et al.^8^ also attributed the lower pH values observed in the deep waters of the AS to the higher water temperature in the deep AS. Further, the higher concentration of dissolved Rare Earth Elements (REE) in the deep AS compared to BoB is attributed to the enhanced vertical mixing^3^.

Recent studies have shown that response to global climate change is more rapid in the deep ocean as well as in the marginal seas than anticipated^5,6^. Tatebe et al.^56^ reported that the tide-induced deep ocean mixing has a significant impact on the climatic mean state in the ocean, as it can regulate the ocean uptake of heat and carbon under the present global climate change scenario. In another study, DeCarlo et al.^57^ showed that internal tide activity is expected to increase in the global warming scenario due to increase in vertical stratification. Hence amplitude of internal waves/tides could be larger in future and such increase could also result in increased internal tide induced mixing. Since the bottom temperature in the deep AS is relatively warmer than the surrounding waters and the vertical mixing is very strong, AS could have a more rapid response to the change in temperature in the global warming scenario compared to other regions. Therefore, modelling and long-term measurements of physical, chemical and biological properties will be crucial in deciphering the long-term change in this region.

## Supplementary information

Supplementary file1 (PDF 322 kb)
