# Supplementary material for: Role of internal tide mixing in keeping the deep Andaman Sea warmer than the Bay of Bengal
Source: Sci Rep. 2020 Jul 20;10:11982. doi: 10.1038/s41598-020-68708-6 (PMC7371704; doi:10.1038/s41598-020-68708-6)
Supplement: Supplementary file 1 — Supplementary file1 (PDF 322 kb) [file 41598_2020_68708_MOESM1_ESM.pdf]

Supplementary information

Role of internal tide mixing in keeping the deep Andaman Sea warmer than the Bay of Bengal

A. K. Jithin* and P. A. Francis
Indian National Centre for Ocean Information Services (INCOIS)
Ministry of Earth Science (MoES), Hyderabad, 500090, India
* jithinoceanography@gmail.com

Supplementary information 1

Comparison of observed and modelled vertical profiles of average temperature in the Bay of Bengal and Andaman Sea.

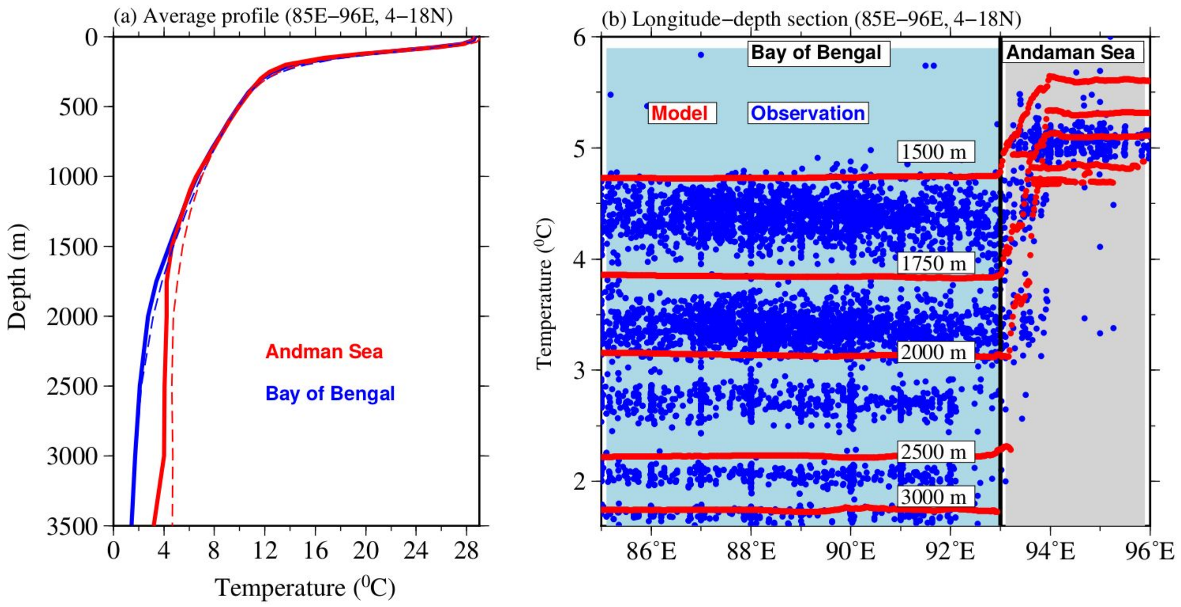

Fig: S1 : (a) Comparison of observed and modelled vertical profiles of average temperature in the Bay of Bengal and Andaman Sea. (b) Longitudinal distribution of observed (blue cirles) and modelled (red circles) temperature in the bay of bengal and Andaman Sea at the bottom levels. Observed and modelled temperatures at 1500, 1750, 2000, 2500 and 3000 m inside 85°-96°E and 4°-18°N are shown here.

Supplementary information 2

To ensure the role of tidal forcing in keeping the deep Andaman Sea warm, we have conducted a longer model simulation with and without tidal forcing. Model is integrated for the period of June 2005 to March 2015 with realistic atmospheric and boundary conditions.

The model configuration and forcing are the same as the control run explained in the main text. Figure S1 shows the time series of average bottom temperature (15000-3750 m) in the Andaman Sea with and without tidal forcing and Figure S2 shows difference between the bottom temperature (1500-37500 m average) with and without tidal forcing after 10 years. Results from these simulations show that the temperature of the bottom layers are warmer (~0.25°C) even after 10 years of simulation with tides compared to without tidal forcing (Figure S1 & S2).

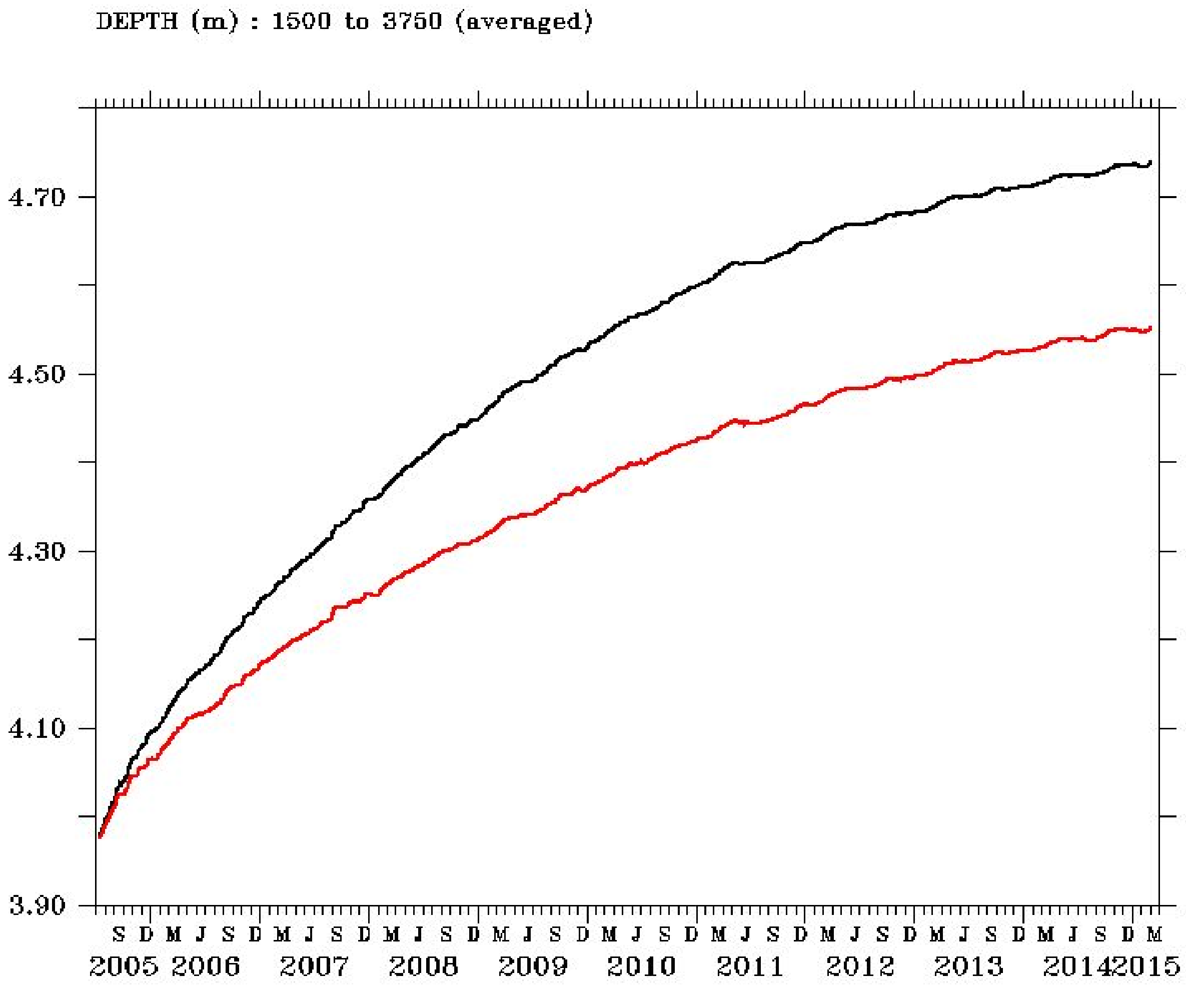

Fig . S1 : Average the bottom temperature (1500-3750 m) with (black line) and without tidal forcing (red line) for 10 years of model simulation.
